# *HSD17B12* dosage insufficiency induced premature ovarian insufficiency in humans and mice

**DOI:** 10.1002/ctm2.737

**Published:** 2022-02-20

**Authors:** Qiqi Wang, Qing Chen, Yixin Zhang, Xue Zhang, Chunyu Liu, Daqi Wang, Yanhua Wu, Yixi Sun, Ling Zhang, Chengcheng Song, Yongming Wang, Yanpeng An, Huiru Tang, Congjian Xu, Yanting Wu, Li Jin, Hefeng Huang, Feng Zhang

**Affiliations:** ^1^ Obstetrics and Gynecology Hospital State Key Laboratory of Genetic Engineering at School of Life Sciences Institute of Reproduction and Development Fudan University Shanghai China; ^2^ NHC Key Laboratory of Reproduction Regulation Shanghai Institute for Biomedical and Pharmaceutical Technologies Fudan University Shanghai China; ^3^ Shanghai Key Laboratory of Female Reproductive Endocrine Related Diseases Shanghai China; ^4^ Department of Reproductive Endocrinology Women's Hospital School of Medicine Zhejiang University Hangzhou China; ^5^ Department of Reproductive Genetics Women's Hospital School of Medicine Zhejiang University Hangzhou China; ^6^ Human Phenome Institute Zhangjiang Fudan International Innovation Center Fudan University Shanghai China

Dear Editor,

1

Premature ovarian insufficiency (POI) is a severe female reproductive disorder that affects 1%−2% of women in general populations.^1^ The analysis of familial POI implies that genetic aberrations strongly influence the onset of POI.^2^ However, a large proportion of POI cases remain idiopathic, suggesting that novel causative or susceptible factors are yet to be discovered.^3^ Here, we identify *HSD17B12* dosage insufficiency as a novel mechanism underlying human POI.

A nonconsanguineous Han Chinese pedigree with two daughters exhibiting POI was investigated (Table S1). The proband (II‐1, Figure 1A) presented primary amenorrhea. Ultrasound examination revealed the infantile uterus (size: 19 × 14 × 18 mm) and invisible ovaries. She was treated with hormone replacement therapy at age 19 to induce menarche and maintain sexual development and cyclical bleeding. Her younger sister (II‐2) experienced normal first menarche at 12 years old. But she was diagnosed with early‐onset POI at 13 years old when occurring amenorrhea. Both sisters have normal 46,XX karyotypes. Their *FMR1* CGG repeat lengths are in the regular polymorphic range. Both parents were reported as healthy with normal high‐resolution karyotypes. The mother had normal pubertal development and continued to have regular menstrual periods at her age of 49. No history of associated endocrinopathies or autoimmune disorders was found in this family.

**FIGURE 1:**
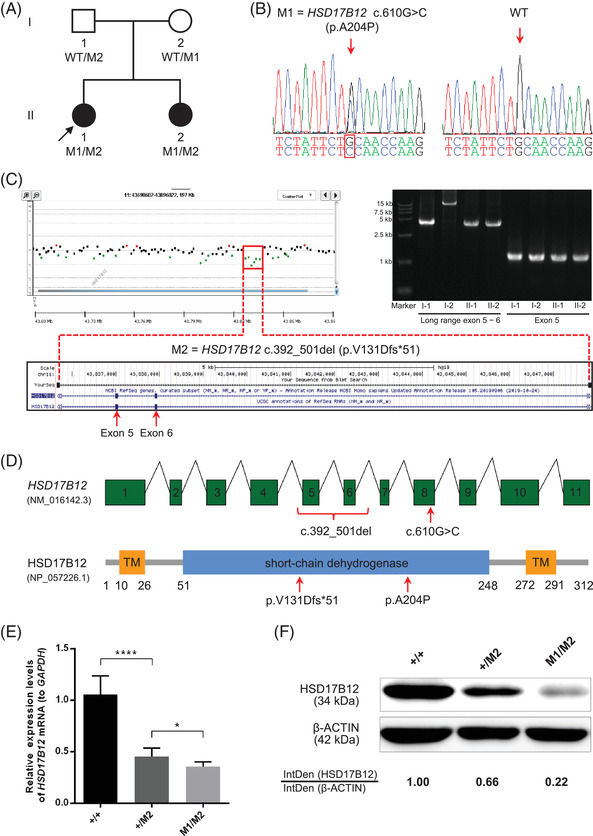
Compound heterozygous variants of *HSD17B12* in the premature ovarian insufficiency (POI) cases from a non‐consanguineous family. (A) Pedigree of the POI family affected by deleterious *HSD17B12* variants. The proband is indicated by a black arrow. Black solid circles indicate the POI cases. WT, wild type; M1 = c.610G > C (p.A204P); M2 = c.392_501del (p.V131Dfs*51). (B) Sanger sequencing confirmed the heterozygous *HSD17B12* variant c.610G > C. The mutated nucleotide is indicated by a red box. (C) The assays of aCGH and long‐range PCR identified a paternally inherited *HSD17B12* exonic deletion (involving exons 5 and 6). The deletion region is noted by a red box. Red arrows indicate the deleted exons. (D) A schematic diagram depiction of *HSD17B12* (top panel) and the protein domains of HSD17B12 (bottom panel). The mutant positions are indicated by red brace and arrows. TM, transmembrane region. Quantitative RT‐PCR (E) and western blotting (F) demonstrated that the relative expression abundances of *HSD17B12* mRNA and protein in the human KGN cells with a heterozygous *HSD17B12* exonic deletion (M2) were approximately half of those in WT controls. Further reductions in the expressions of *HSD17B12* mRNA and protein were revealed in the KGN cells with bi‐allelic *HSD17B12* variants (M1 + M2). *GAPDH* and β‐ACTIN were used as internal controls of quantitative RT‐PCR and western blotting, respectively. Data were presented as mean ± standard deviation of three independent experiments. Statistical significance (Student's *t*‐test): *p < .05; ****p < .0001. Band density was quantified using ImageJ. Representative images of three independent experiments are shown. IntDen, integrated density

We conducted whole‐exome sequencing and subsequent array‐based comparative genomic hybridization (aCGH) and found that both index POI sisters harbored compound heterozygous variants in *HSD17B12* (Figure 1A), including a novel missense variant (M1: c.610G > C, p.A204P) and a novel deletion (M2). The maternally inherited missense variant (Figure 1B) was confirmed by Sanger sequencing (Table S2) and predicated to be pathogenic by bioinformatic tools (Table 1). Additionally, long‐range polymerase chain reaction (PCR) confirmed a paternally inherited deletion affecting *HSD17B12* exons 5 and 6 (Figure 1C).

**TABLE 1:** Summary of compound heterozygous HSD17B12 variants identified in this study

**Variant and inheritance**		
Nucleotide change^a^	Chr11:43859940G>C	Chr11:43835725‐43847793del
Parent of origin	Maternal	Paternal
Mutation type	Missense	Exonic deletion, frameshift
cDNA mutation^b^	c.610G>C	c.392_501del
Protein alteration	p.A204P	p.V131Dfs*51
**Minor allele frequency** **^c^**		
1KGP	0	N/A
gnomAD	0	N/A
DGV	N/A	0
**Functional prediction** **^d^**		
SIFT	Damaging	N/A
PolyPhen‐2	Probably damaging	Damaging
MutationTaster	Disease causing	Damaging
CADD	6.790861	N/A
DANN	.998	N/A
American College of Medical Genetics and Genomics classification	Pathogenic	Pathogenic
**Conservation** **^e^**		
PhyloP	4.589	N/A
PhastCons	1	N/A

Abbreviations: N/A, not applicable.

^a^ Genomic coordinates of the human genome assembly GRCh37/hg19.

^b^ The NCBI accession number of *HSD17B12* is NM_016142.3.

^c^ According to the 1000 Genomes Project (1KGP), Genome Aggregation Database (gnomAD), and Database of Genomic Variants (DGV).

^d^ See Supplemental Information for details. High CADD and DANN scores usually indicate that variants are likely to have deleterious effects. CADD cutoff is usually set at 4, and DANN cutoff is set at .93.

^e^ A positive phyloP score and a phastCons value close to 1 indicate a predicted conserved nucleotide.

*HSD17B12* (*hydroxysteroid 17‐beta dehydrogenase 12*) encodes a major enzyme responsible for the estrone to estradiol (E2) conversion.^4^ Importantly, the A204 position of HSD17B12 is conserved according to phyloP and phastCons programs (Table 1), and the p.A204P variant could disturb the structural stability of HSD17B12 by missing hydrogenbonds formed between A204 and other amino acids (Figure S1). Additionally, both *HSD17B12* missense and deletion variants carried by both index sisters are located in the crucial short‐chain dehydrogenase/reductase domain (Figure 1D) that is conserved during evolution.^5^ Dramatically, no obvious HSD17B12 was detected in the blood samples of index sisters (Figure S2), indicating deleterious effects of *HSD17B12* variants.

To further investigate the pathogenesis of bi‐allelic *HSD17B12* variants in POI, we knocked these variants into human ovarian KGN cells using CRISPR‐Cas9 system (Table S2). Firstly, we sequenced the low‐abundance complementary DNA of *HSD17B12* ^M2/M2^ KGN cells and revealed that the M2 deletion of *HSD17B12* exons 5 and 6 (c.392_501del) created a frameshift in canonical HSD17B12 (NP_057226.1: p.V131Dfs*51) (Figure S3). Secondly, quantitative reverse transcription PCR (RT‐PCR) demonstrated that *HSD17B12* ^+/M2^ KGN cells expressed only a half abundance of *HSD17B12* when compared with wild‐type (WT) controls (Figure 1E), suggesting the possibility of nonsense‐mediated mRNA decay.^6^ Thirdly, we observed a further reduced expression of *HSD17B12* in *HSD17B12* ^M1/M2^ KGN cells (Figure 1E,F). The above experimental evidence suggested a gene dosage effect caused by the combination of paternally and maternally inherited deleterious variants in *HSD17B12*.

Furthermore, we generated gene‐edited mouse models to investigate in vivo function of *Hsd17b12* (Figure S4). We crossed *Hsd17b12* ^+/−^ and *Hsd17b12* ^+/A204P^ mice of 8 weeks old. However, no *Hsd17b12* ^−/A204P^ mice were found at birth (Table S3), which was due to the embryogenic lethality at E11.5. Coincidentally, we failed to obtain *Hsd17b12* ^−/−^ or *Hsd17b12* ^A204P/A204P^ live mice by intercrossing heterozygous mutants, since they died at E8.5 and P0, respectively (Table S3). These experimental observations indicate that mice are more vulnerable than human subjects to *HSD17B12*/*Hsd17b12* dosage insufficiency. These divergent phenotypes between bi‐allelic *HSD17B12*/*Hsd17b12‐*mutated humans and mice might be attributed by that the 17b‐hydroxysteroid dehydrogenases are largely multifunctional enzymes, and their functions depend on the substrates in different species or the isoenzymes.^7^

Since bi‐allelic *Hsd17b12‐*mutated mice were embryonically lethal, heterozygous *Hsd17b12*‐mutated adult mice were recruited to determine the effects of *Hsd17b12* variants on female fertility. Although there was no significant difference in ovarian index between heterozygous *Hsd17b12*‐mutated and WT groups (Table S4), significantly decreased mRNA abundances of *Hsd17b12* were observed in the ovaries of *Hsd17b12* ^+/−^ and *Hsd17b12* ^+/A204P^ female mice when compared with their WT littermates (Figure 2A). Subsequently, *Hsd17b12* ^+/−^ and *Hsd17b12* ^+/A204P^ female mice of 8 weeks old and their WT control littermates were mated with WT adult males for up to 6 months. Surprisingly, although the offspring fit a conventional pattern of Mendelian inheritance (Table S5), both *Hsd17b12* ^+/−^ and *Hsd17b12* ^+/A204P^ female mice showed significantly longer times between pregnancies and smaller litter sizes than WT controls (Table 2). Additionally, after 2 months daily examination of estrus cycle, *Hsd17b12* ^+/−^ female mice showed inordinate prolongations in the metestrus and diestrus and so that the entire estrous cycle when compared to WT controls (Figure 2B,C). To further visualize the potential female subfertility, the morphologies of follicles at different developmental stages were examined by hematoxylin and eosin staining. Both *Hsd17b12* ^+/−^ and *Hsd17b12* ^+/A204P^ female mice showed significantly reduced numbers of developing‐follicles in their ovaries when compared to WT controls (Figure 2D), indicating the correlation between *Hsd17b12* dosage insufficiency and female subfertility in mice.

**FIGURE 2:**
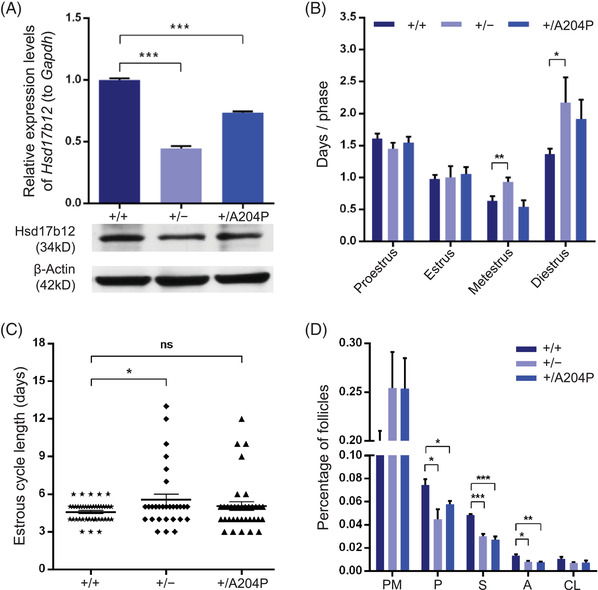
Dosage insufficiency of *Hsd17b12* affected the fertility of female mice. (A) Both *Hsd17b12* ^+/−^ and *Hsd17b12* ^+/A204P^ female mice (14 weeks old) showed significantly reduced *Hsd17b12* mRNA expressions when compared with wile‐type female mice. (B) Metestrus and diestrus of estrous cycle of *Hsd17b12* ^+/−^ female mice were significantly longer than those of wile‐type female controls. (C) The entire estrous cycles of *Hsd17b12* ^+/−^ female mice were significantly longer than those of wile‐type female controls. (D) Follicle ratio counting was performed in the ovaries of 14‐week‐old female mice. Follicle ratio refers to the percentage of the follicle number at a specific developmental stage to the total follicle number. A, antral follicles; CL, corpus luteum; ns, not significant; P, primary follicles; PM, primordial follicles; S, secondary follicles. Statistical significance: *p < .05; **p < .01; ***p < .001

**TABLE 2:** Affected fertility of Hsd17b12‐mutated female mice

Genotype	Number of females	Days between pregnancies/litters	Mean of litter size
Wild type	14	22.8	8.1
*Hsd17b12^+/−^*	13	32.6****	7.0*
*Hsd17b12* ^+/A204P^	10	29.8****	7.1*

*Note*: Student's *t*‐test when compared with the wild‐type.

* *p* < .05.

**** *p* < .0001.

As for *Hsd17b12* homozygous mutants, *Hsd17b12* ^A204P/A204P^ mice lived till P0; therefore, the P0 female mice were used for three‐dimensional structure reconstruction of cleared‐ovaries, which showed smaller and thinner ovaries, and consistently, fewer primordial follicles in *Hsd17b12* ^A204P/A204P^ female mice than those in WT controls (Figure S5), further suggesting that *Hsd17b12* is essential for ovarian morphology and reserve.

HSD17B12 was previously reported in the regulation of female reproduction potentially via the metabolism of arachidonic acid, a precursor of prostaglandins.^8^, ^9^, ^10^ Coincidently, the metabolic processes of arachidonic acid and related products are affected in the ovaries of heterozygous *Hsd17b12*‐mutated female mice (Figure S6).

In conclusion, our observations based on human POI subjects, gene‐edited KGN cell, and mouse models supported the pathogenic roles of *HSD17B12* deleterious variants and their associated HSD17B12 dosage insufficiency in POI. *HSD17B12* is genetically involved in human early‐onset POI and even primary amenorrhea via the autosomal recessive inheritance.

## CONFLICT OF INTEREST

The authors declare that they do not have any commercial or associative interest that represents a conflict of interest in connection with the work submitted.

## Supporting information

Supporting information
