# Supplementary material for: HSD17B12 dosage insufficiency induced premature ovarian insufficiency in humans and mice
Source: Clin Transl Med. 2022 Feb 20;12(2):e737. doi: 10.1002/ctm2.737 (PMC8858626; doi:10.1002/ctm2.737)
Supplement: Supplementary file 1 — Supporting information [file CTM2-12-e737-s001.pdf]

SUPPLEMENTAL METHODS

Human subjects

Two cases with premature ovarian insufficiency (POI) from a nonconsanguineous Han Chinese family (Figure 1A) were recruited at the Women's Hospital of Zhejiang University, China. All female participants were clinically assessed by ultrasound examination and hormone levels.

Whole-exome sequencing (WES) and bioinformatic analysis

WES was performed for the family according to the protocol we described previously.[1] Briefly, genomic DNA was extracted from peripheral blood using a Puregene Blood Core Kit B (Qiagen) according to standard procedures, and then used to enriched exome by a SureSelect^{XT} Human All Exon V6 kit (Agilent Technologies). The library was sequenced on Illumina HiSeq X-TEN platform at Cloud Health Genomics (Shanghai, China). Raw data of approximately 10 GB was mapped and aligned to the human reference genome (GRCh37/hg19) using Burrows–Wheeler Aligner tool.[2] Polymerase chain reaction (PCR) duplicate removing and variant qualifying were operated using Picard software (http://picard.sourceforge.net). Genome Analysis Toolkit[3] and ANNOVAR software were further used for variant calling and annotating, respectively. Minor allele frequencies of variants in human were estimated using the 1000 Genomes Project (1KGP) via the Ensembl database (www.ensembl.org) and the Genome Aggregation Database (gnomAD; http://gnomad-sg.org). SIFT (https://sift.bii.a-star.edu.sg), PolyPhen-2

(http://genetics.bwh.harvard.edu/pph2/), MutationTaster (http://www.mutationtaster.org), Combined Annotation Dependent Depletion (CADD; https://cadd.gs.washington.edu/snv), DANN[4] and American College of Medical Genetics and Genomics (ACMG) classification[5] were employed for preliminary predictions of deleterious variants. The conservations of mutant sites were estimated by phyloP[6] and the phastCons[7] scores.

Array-based comparative genomic hybridization (aCGH) analysis

To investigate whether there were causative deletion or duplication SVs for POI, the index sisters were subjected to SurePrint G3 Human 1×1M CGH Microarrays (Agilent, Santa Clara, USA). Some experimental details were previously described.[8] In brief, 2 μg of test DNA and gender-matched reference DNA were double-digested with *Rsa*I and *Alu*I (Promega) for 1 hour at 37°C and subsequently labeled with Cy5 and Cy3-dUTP using the Agilent SureTag DNA Labeling Kit, respectively. Purification of labeling products, array hybridization, washing, scanning, and data analysis were conducted by following the Agilent aCGH protocol (version 6.0). The Database of Genomic Variants (DGV; http://dgv.tcag.ca/dgv/app/home) was used to compare findings to previously reported studies.[9] Coordinates of structural variants (SVs) are based on the human genome reference assembly GRCh37/hg19.

Sanger sequencing

Variants identified in exome sequencing were validated using Sanger sequencing. The

approximate positions of the SV breakpoints in genome were preliminarily pinpointed by aCGH data. Long-range PCR conducted with PrimeSTAR® GXL DNA Polymerase (TaKaRa) was performed to amplify the deletion breakpoint. Primers for pedigree analysis in this family are presented in Table S2. DNA sequences were aligned to the human genome reference assembly (GRGh37/hg19) with the BLAT tool from the UCSC Genome Browser.

Generation of cell models using CRISPR-Cas9

To experimentally assess the functional consequences of the *HSD17B12* variants identified in our study, we deleted the 5th and 6th exons of the *HSD17B12* gene (M2 in Figure 1) in human KGN cells using the epiCRISPR system.[10] Two single guide RNAs (sgRNAs) neighbored the breakpoint of *HSD17B12* were designed using Zhang Lab Guide Design Resources (http://crispr.mit.edu/) and GPP Web Portal (http://portals.broadinstitute.org/gpp/public/) (P1-1F and P2-1R in Table S2). The epiCRISPR_gRNA1_gRNA2_vector, contained a respective U6 promoter neighbored the 5′ of each gRNA, was successfully established and then transfected into KGN cells using lipofectamine 3000 Reagents (Life Technologies) according to the manufacturer's instruction. After 24 hours of transfection, cells cultured and selected for 1 week with 0.4 μg/ml Puromycin. Cells expressing EGFP were separated with flow cytometry and plated on dishes. Single colonies were picked and expanded for genotyping using long range PCR primers to confirm the presence of cells with M2.

Then, the pX458 plasmid harboring corresponding sgRNA targeted *HSD17B12*

missense mutation c.610G>C (M1 in Figure 1) (KI-1F and KI-1R in Table S2), were co-transfected into *HSD17B12*$^{+/M2}$ cells with homologous arm vectors, which finally generated cell line with bi-allelic *HSD17B12* variants that mimicked the index patients.

Quantitative reverse transcription PCR (RT-PCR) and western blot

Total RNA and proteins were extracted from KGN cells using a AllPrep DNA/RNA/Protein Mini Kit (Qiagen) according to the manufacturer's protocol. Reverse-transcription reactions were carried out using 1μg total RNA. Relative mRNA levels of *HSD17B12* were quantified using special primers pairs (Table S2) and SYBR-Green PCR kit (Qiagen). RNA expression levels were normalized to the basal level of human *GAPDH* (internal control) using the $2^{-\Delta\Delta Ct}$ method. At least three samples for each genotype were analyzed. Quantitative RT-PCR reactions were done in triplicate for each experiment. All statistical tests were carried out using the GraphPad Prism statistical analysis software version 6.0 (GraphPad Software, CA, USA).

Protein lysates were prepared and separated on a 10% sodium dodecyl sulfate polyacrylamide gel electrophoresis (SDS-PAGE) and transferred onto polyvinylidene difluoride membrane (Immobilon-P, Merck Millipore, USA). Then the membranes were blocked with 10% skimmed milk (Nacalai tesque, Japan) and incubated with rabbit anti-HSD17B12 (#ab103107, Abcam, UK) antibodies overnight at 4°C, followed by horseradish peroxidase (HRP)-conjugated secondary antibodies (#111-036-045, Jackson ImmunoResearch, USA) for 2 hours at room temperature. Detection was carried out with

Chemi-Lumi One Super (Nacalai tesque).

Generation of mouse models using CRISPR-Cas9

C57BL/6 mouse model with point mutation (c.610G>C) at *Hsd17b12* (NM_019657.4) was generated using the CRISPR-Cas9 technology (BIOGLE, China). Cas9 mRNA and corresponding sgRNA (sgRNA and ssODN in Table S2) transcribed in vitro were co-injected into the zygotes for knock-in mouse production.[11] The treated zygotes were cultured in KSOM medium[12] to the two-cell stage and transplanted into the oviducts of pseudopregnant foster females at 0.5 day after mating with vasectomized males. After identifying the genotypes though Sanger sequencing, founder mice with c.610G>C mutations or frameshift mutations (F0) were bred with wild-type C57BL/6 mice to obtain enough *Hsd17b12*-mutant pups (F1). Then, *Hsd17b12* mutated F1 mice underwent serial mating to generate homozygous and compound heterozygous offspring.

Animal studies were approved by the institutional review board at Fudan University and were carried out in accordance with the recommendation in the Guide for the Care and Use of Laboratory Animals of the US National Institutes of Health. The mice were bred in specific pathogen-free barrier facility. C57BL/6J was the strain background used in all mouse experiments.

Assessment of embryo lethal time

To investigate the lethal time of *Hsd17b12*-deficient embryos, *Hsd17b12*$^{+/A204P}$ and

Hsd17b12$^{+/-}$ mice were self- or cross- mated when they were 8 weeks old. Couple mice were caged together at night and separated before 9 am the next day to check the vaginal plug. Female mice with vaginal plugs were removed and isolated, and correspondingly, the embryos were recorded as embryonic day (E) 0.5. Genomic DNA of the yolk sack enclosed E10.5 to postnatal day (P) 0 were obtained using a DNeasy Blood & Tissue Kit (Qiagen). DNA from target region was amplified and the mutations were verified by Sanger sequencing.

Fertility analysis of female mice

Female mice were divided into three equal groups according to their genotypes, and the 15 female mice (8 weeks old) in each group were mated with 15 wild-type males respectively for 6 months, followed by recording litter sizers and the frequency of litters born. Also, the genotype and the gender of pups were counted.

Estrous cycles of adult female mice

Estrous cycles of 30 adult females of each genotype were then assessed for 4 weeks. Vaginal cells from females aged 2 to 3 months were collected by daily saline washes and analyzed after Wright-Giemsa staining. Stages of the estrous cycle were characterized by predominant nucleated cells, predominant cornified epithelial cells and predominant leukocytes for proestrus (P), estrus (E), metestrus (M) and diestrus (D) stages, respectively.

Analyses of ovarian histology and metabolomics

Female mice of age 12-weeks were sacrificed when they were in diestrus. Mice were anesthetized with isoflurane to collect the blood by hepatic portal vein puncture. Left ovaries were immediately excised and stored at −80°C for metabolomics analysis, and right ovaries were fixed in 10% neutral formalin fix solution (Sangon Biotech, Shanghai, China) for further histological studies. Blood samples were allowed to clot at 37℃ for 20 minutes, and then centrifuged at 10000 rpm for 5 min to obtain the serum. For metabolomics analysis, the metabolites including arachidonic acid (AA), prostaglandin F1α (6-keto PGF1α), PGE2, PGF2α and other oxidized polyunsaturated fatty acids were examined by HPLC-tandem mass spectrometry.[13,14] Microscopic analysis and follicle counting were performed as previously reported.[1]

Tissue clearing and ovarian reserve analysis

Ovaries of P0 female mice were collected in phosphate-buffered saline (PBS) and then fixed in 4% paraformaldehyde overnight at 4°C. Ovary clearing were operated based on the work of Safia Malki et al.,[15] and a combination of anti-P63 antibody (Abcam, ab53039) and anti-Tra98 antibody (Abcam, ab82527) was used to identify primordial follicles. All of the cleared ovaries were then imaged by Nikon A1 confocal microscope (Japan) with a 10× NA 0.5 dry objective, which scanned with step size of 3 μm for the Z stack to stitched a three-dimensional (3D) structural image for further analysis. Imaris software (v9.3.1) was used to analyze the 3D image.

Oocytes were identified by spot transformation after manual correction. The principal parameters were set and adjusted colligating oocyte size, object quality (minimum score=100) and the intensity of the signal. Errors in the software detection results were corrected by manual correction.

Statistical analysis

Effects of genotype on gene expression levels in adult mice were analyzed by unpaired, two-tailed *t* tests. All data are presented as mean ± SEM. P <0.05 was considered statistically significant. Statistical analysis was performed using the GarphPad Prism5 version 5.01 software (GraphPad Software, San Diego, CA, USA). The unpaired *t-test* procedure was used to compare weight between wild-type and homozygous ovarian reverses.

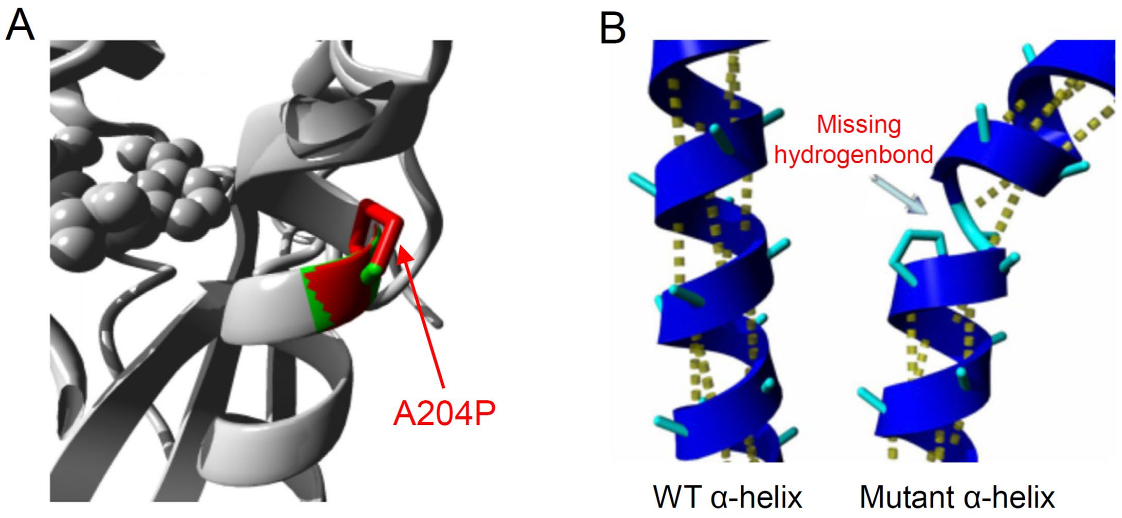

FIGURE S1 Functional prediction of the A204P variant in HSD17B12. (A) The structural prediction of wild-type (WT) and A204P-mutated HSD17B12. A merged image was created by HOPE software. The mutant amino acid is indicated by a red arrow. The protein is colored grey, and the side chains of both WT and mutant residues are colored by green and red, respectively. (B) Three-dimensional structure of HSD17B12 A204P variant. This variant results in missing hydrogenbond into the α-helix, and severely affects the structure of HSD17B12 protein. Residues and hydrogenbonds are colored by light blue and yellow-green, respectively.

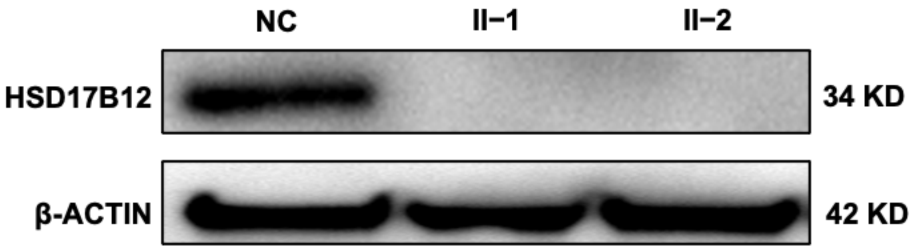

FIGURE S2 *HSD17B12* compound heterozygous variants affect HSD17B12 protein expressions. The GTEx database indicates the expression of *HSD17B12* in blood cells. Therefore, fresh blood samples were obtained from the index sisters (II-1 and II-2) and three unrelated normal control (NC) women for protein isolation and evaluation of HSD17B12 expression. No obvious HSD17B12 expression was detected in the blood samples from subjects II-1 and II-2 with *HSD17B12* compound heterozygous variants. β-ACTIN was used as an internal control of western blotting.

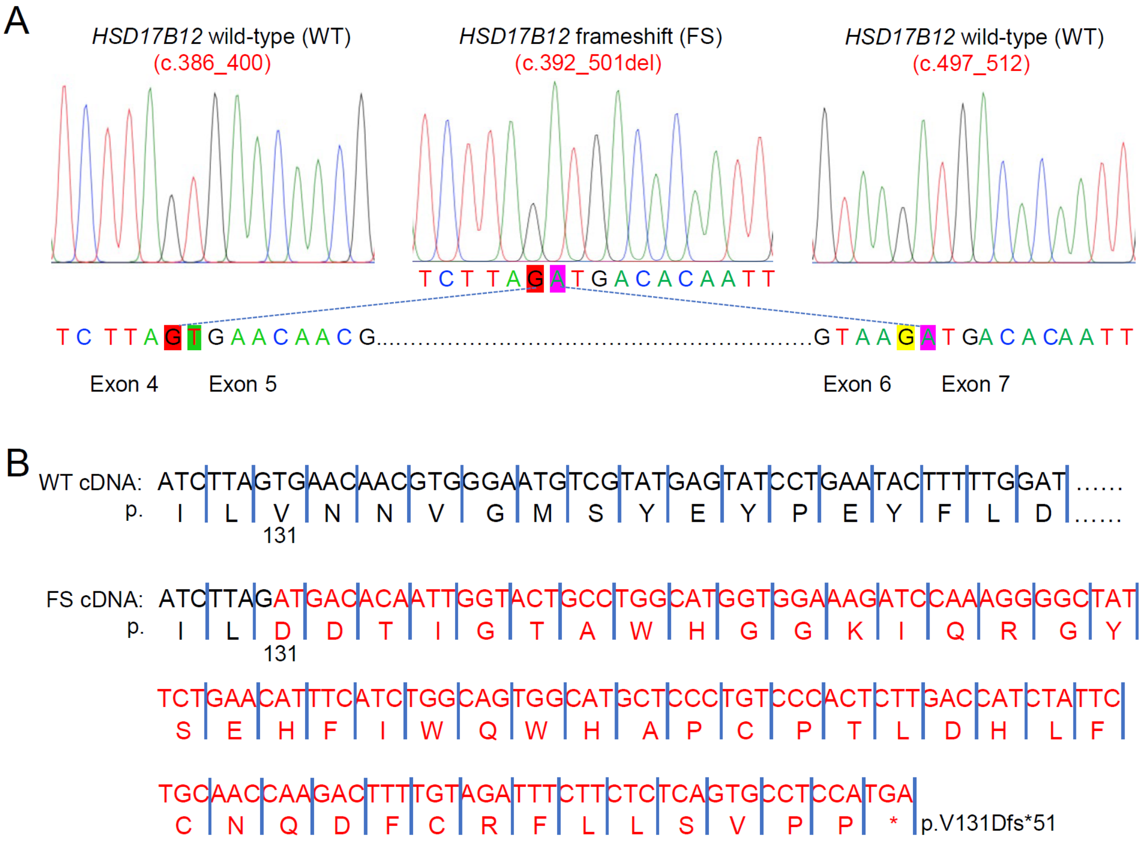

FIGURE S3 The deletion of *HSD17B12* exons 5 and 6 leads to a frameshift variant in human *HSD17B12*. (A) KGN cells with an exonic deletion (exons 5 and 6) of *HSD17B12* (NM_016142.3) was generated using CRISPR-Cas9 technology. The last nucleotides of exons 4 and 6 are marked by red and yellow, respectively. The first nucleotides of exons 5 and 7 are marked by green and pink, respectively. The deleted nucleotide sequence is enclosed by blue dashes. (B) The frameshift variant leads to a premature translational termination (p.V131Dfs*51) of HSD17B12 (NP_057226.1). The asterisk (*) indicates the termination code.

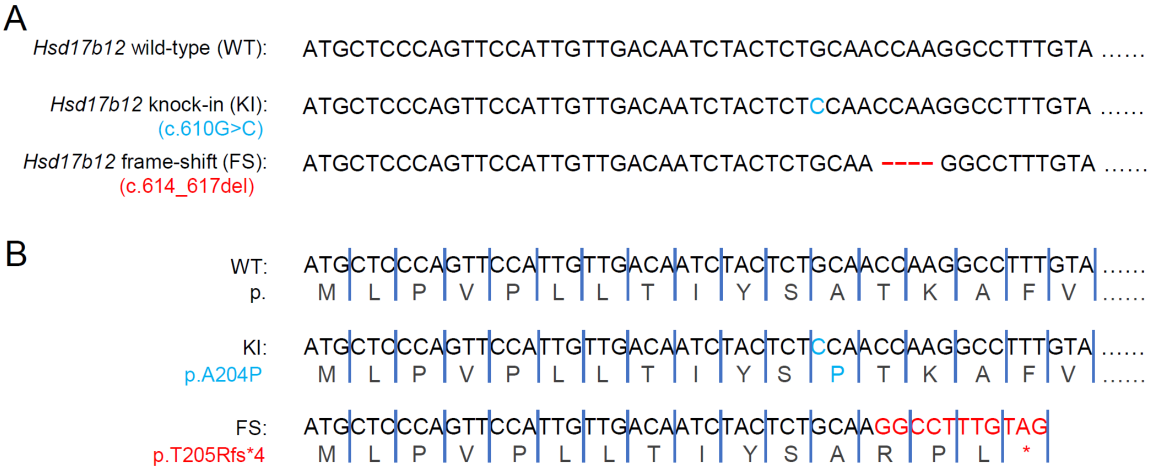

FIGURE S4 Genotypes of two *Hsd17b12* mutants in mice. (A) The knock-in mouse model (c.610G>C) and knock-out mouse model (c.614_617del, frameshift) of *Hsd17b12* (NM_019657.4) were generated using CRISPR-Cas9 technology. The mutated nucleotide is marked by light blue, and the deleted sequence is shown by red dashes. (B) The c.610G>C variant in *Hsd17b12* leads to a missense mutation (p.A204P) in Hsd17b12 (NP_062631.1), and the c.614_617del variant leads to a premature translational termination (p.T205Rfs*4) of Hsd17b12. The asterisk (*) indicates the termination code.

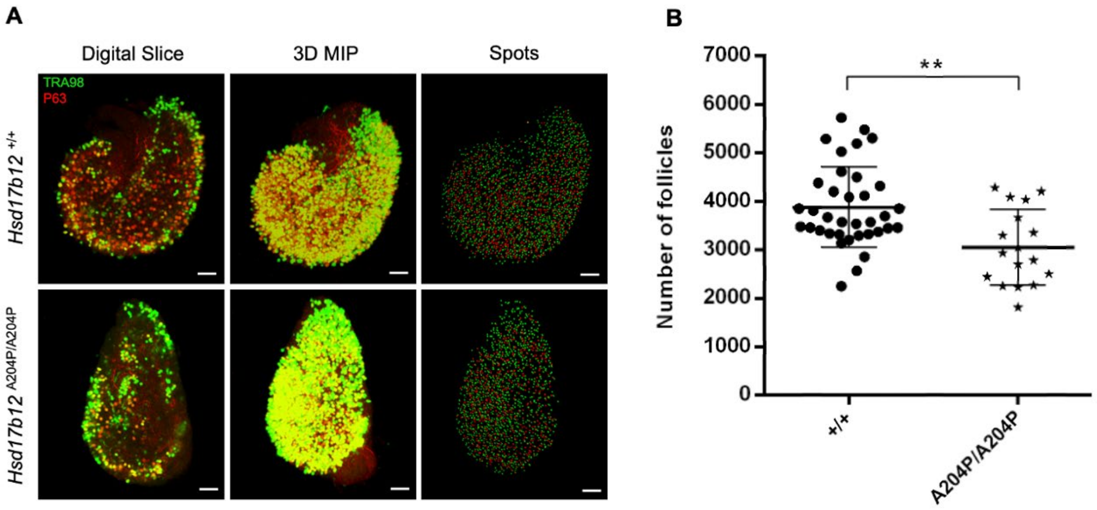

FIGURE S5 Affected ovarian development in newborn *Hsd17b12*$^{A204P/A204P}$ female mice. (A) Three-dimensional structure reconstruction of cleared ovaries. *Hsd17b12*$^{A204P/A204P}$ female mice, which died within several hours of birth, presented abnormal ovarian morphologies. Oocyte nuclei were stained by TRA98 (green) and P63 (red). Spot transformation was used to identify positive nuclei. Green spots, TRA98 positive oocytes; red spots, P63 positive oocytes; and yellow spots, TRA98 and P63 co-expressed oocytes. MIP, maximum intensity projection. Scale bar: 75 µm. (B) Counting primordial follicles in cleared ovaries. Statistical significance: ** $P < 0.01$.

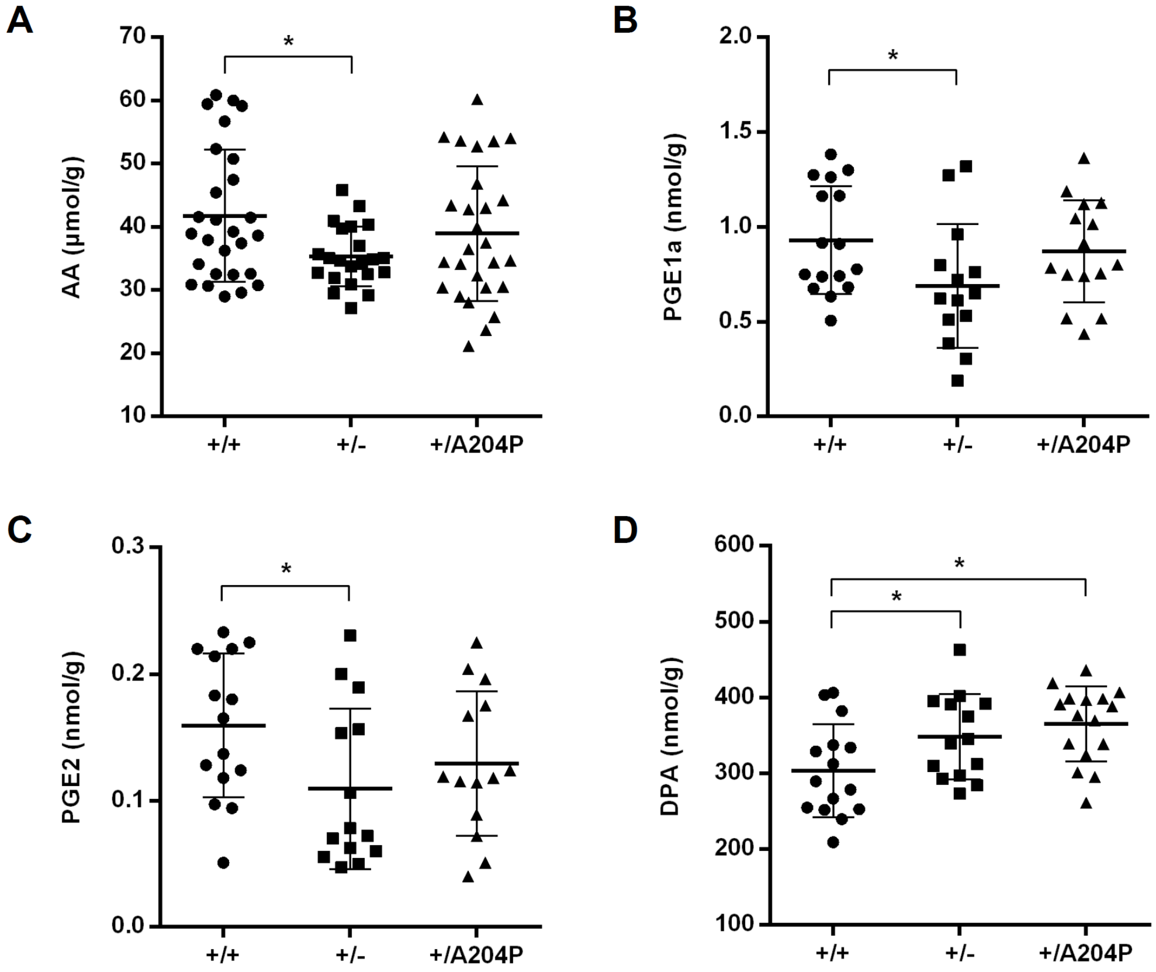

FIGURE S6 Levels of arachidonic acid pathway metabolites in 14-week-old mouse ovaries. (A−C) The levels of arachidonic acid (AA), PGE_{1a} and PEG_2 prostanoids in the ovaries of *Hsd17b12*$^{+/-}$ female mice were significantly lower than those in wild-type (*Hsd17b12*$^{+/+}$) controls. (D) Docosapentaenoic acid (DPA) levels in the ovaries of both *Hsd17b12*$^{+/-}$ and *Hsd17b12*$^{+/A204P}$ female mice were significantly higher than those in wild-type controls. Statistical significance: * $P < 0.05$.

TABLE S1 Clinical characteristics of the POI subjects affected by compound heterozygous *HSD17B12* variants

	**Subject**	
**Characteristic**	**II-1**	**II-2**
First menses (years)	PA	12
Age of POI (years)	PA	13
Weight (kg)	48	52
Height (cm)	157	160
FSH (IU/L)	123.3	98.0
LH (IU/L)	26.3	30.4
PRL (ng/mL)	11.8	NA
E2 (pg/mL)	28.9	< 18.3
T (ng/mL)	1.3	NA
Ovarian size (right/left) (mm)	Invisible	NA
Follicle per ovary (right/left) (n)	Not obvious	NA
Karyotype	46,XX	46,XX
FMR1 CGG repeats (n)	30/39	30/39

Abbreviations: E2, estradiol; FSH, follicle-stimulating hormone; LH, luteinizing hormone; NA, not available; PA, primary amenorrhea; PRL, prolactin; T, testosterone.

TABLE S2 Sequence information of primers and oligos

Assay		Primer/oligo sequence (5′ − 3′)	Product length (bp)
Human case	Long range PCR	TGCTTGCTTAGTTATTCAGGGACTTCC<br>GACATAAACACTGATTTCAATGCCTGG	16098
	Missense variant genotyping	ACTGATGAAAGGCAGGGAGAGA<br>GGGGAGAAAAACAGCATCTGTG	480
KGN cell	P1-1F[a]	CGCTCTTCGCCG**GTAATATTCCCATAGGC TAG**gttttagagctagaaatagcaa	
	P2-1R[a]	CGCTCTTCTAAC**GTAGCGTCAGTAATCAA CAC**cggtgtttcgtcctttccac	
	KI-1F	TGCTTGCTTAGTTATTCAGGGACTTCC	
	KI-1R	GACATAAACACTGATTTCAATGCCTGG	
	Genotyping	CACTCTTGACCATCTATTCTC<br>GTCTATCTTGTGCCTGCTAT	483
	qRT-PCR	ATTGGTACTGCCTGGCATGG<br>CAAAGACGCCCTTGCTCCTA	170
	GAPDH control	ATGTTCGTCATGGGTGTGAA<br>GTTGTCATGGATGACCTTGG	114
Mouse model	sgRNA	GACAATCTACTCTGCAACCA	
	ssODN	CTTATGTTGACATAATTTACTGCAGTGTTCG<br>TTAGTCACCTTGGTTGGAGAGTAGATTGTC<br>AACAATGGAACTGGGAGCATGCCACTG G	
	Genotyping	CTGTGACACACTGGCACTCC<br>ATTCACCGGCCAGACATACC	427
	qRT-PCR	CCTGGCATGGTAGAAAGA<br>GAGGCACTGAGAGAAGAAATC	129
	Gapdh control	TAACATCAAATGGGGTGAGG<br>GGTTCACACCCATCACAAAC	161

[a] Single guide RNAs (sgRNA) for genomic deletion using the epiCRISPR system. The gRNA1 sequence and gRNA2 reverse complement sequence are indicated in bold. The lowercase sequences are combined with tracrRNA and human U6 promoter respectively to amplify tracr-U6 from an intermediate plasmid (gRNAU6).

TABLE S3 Genotype analysis of live births in mice

Mating	Litters	*Hsd17b12* genotype Number (%) of live births			
$Hsd17b12^{+/-}$ * $Hsd17b12^{+/-}$	21	+/+	+/−		−/−
		42 (31)	94 (69)		0 (0) [a]
$Hsd17b12^{+/A204P}$ * $Hsd17b12^{+/-}$	31	+/+	+/−	+/A204P	A204P/−
		55 (33)	58 (34)	56 (33)	0 (0) [a]
$Hsd17b12^{+/A204P}$ * $Hsd17b12^{+/A204P}$	47	+/+	+/A204P		A204P/A204P
		105 (35)	194 (65)		0 (0) [a]

[a] Three genotypes of *Hsd17b12*-mutated mice (−/−, A204P/−, and A204P/A204P) are embryogenically lethal.

TABLE S4 Ovary indexes for female mice

**Genotype**	**Body weight (g)**	**Ovary weight (mg)**	**Ovary index (100%*mg/g)**
WT	20.24 ± 0.26 (N=21)	7.45 ± 0.29	0.37
Hsd17b12$^{+/-}$	21.10 ± 0.37 (N=14)	8.05 ± 0.30	0.38
Hsd17b12$^{+/A204P}$	20.93 ± 0.28 (N=15)	7.29 ± 0.24	0.35

Note: Comparison of female body and ovarian weights in diestrus stage during 14 − 18 weeks in mice of wild-type (WT), +/−, and +/A204P genotypes. Values represent the mean ± SEM; statistical analyses were conducted using one-way ANOVA.

TABLE S5 Gender statistics of live births in mice

Mating	Litters	Number (%) of live births	
		Female (%)	Male (%)
Hsd17b12$^{+/-}$ * *Hsd17b12*$^{+/-}$	21	67 (50)	69 (50)
Hsd17b12$^{+/A204P}$ **Hsd17b12*$^{+/-}$	31	88 (52)	81 (48)
Hsd17b12$^{+/A204P}$ * *Hsd17b12*$^{+/A204P}$	47	158 (53)	141 (47)

SUPPLEMENTAL REFERENCES

1. Chen Q, Ke H, Luo X, et al. Rare deleterious BUB1B variants induce premature ovarian insufficiency and early menopause. *Hum Mol Genet.* 2020;29(16):2698-2707.
2. Li H, Durbin R. Fast and accurate long-read alignment with Burrows-Wheeler transform. *Bioinformatics.* 2010;26(5):589-595.
3. McKenna A, Hanna M, Banks E, et al. The Genome Analysis Toolkit: a MapReduce framework for analyzing next-generation DNA sequencing data. *Genome Res.* 2010;20(9):1297-1303.
4. Quang D, Chen Y, Xie X. DANN: a deep learning approach for annotating the pathogenicity of genetic variants. *Bioinformatics.* 2015;31(5):761-763.
5. Richards S, Aziz N, Bale S, et al. Standards and guidelines for the interpretation of sequence variants: a joint consensus recommendation of the American College of Medical Genetics and Genomics and the Association for Molecular Pathology. *Genet Med.* 2015;17(5):405-424.
6. Pollard KS, Hubisz MJ, Rosenbloom KR, Siepel A. Detection of nonneutral substitution rates on mammalian phylogenies. *Genome Res.* 2010;20(1):110-121.
7. Siepel A, Bejerano G, Pedersen JS, et al. Evolutionarily conserved elements in vertebrate, insect, worm, and yeast genomes. *Genome Res.* 2005;15(8):1034-1050.
8. Boone PM, Bacino CA, Shaw CA, et al. Detection of clinically relevant exonic copy-number changes by array CGH. *Hum Mutat.* 2010;31(12):1326-1342.
9. MacDonald JR, Ziman R, Yuen RK, Feuk L, Scherer SW. The Database of Genomic

Variants: a curated collection of structural variation in the human genome. *Nucleic Acids Res.* 2014;42(Database issue):D986-992.

10. Xie Y, Wang D, Lan F, et al. An episomal vector-based CRISPR/Cas9 system for highly efficient gene knockout in human pluripotent stem cells. *Sci Rep.* 2017;7(1):2320.
11. Ittner LM, Gotz J. Pronuclear injection for the production of transgenic mice. *Nat Protoc.* 2007;2(5):1206-1215.
12. Ho Y, Wigglesworth K, Eppig JJ, Schultz RM. Preimplantation development of mouse embryos in KSOM: augmentation by amino acids and analysis of gene expression. *Mol Reprod Dev.* 1995;41(2):232-238.
13. Rantakari P, Lagerbohm H, Kaimainen M, et al. Hydroxysteroid (17{beta}) dehydrogenase 12 is essential for mouse organogenesis and embryonic survival. *Endocrinology.* 2010;151(4):1893-1901.
14. Kemilainen H, Adam M, Maki-Jouppila J, et al. The Hydroxysteroid (17beta) Dehydrogenase Family Gene HSD17B12 Is Involved in the Prostaglandin Synthesis Pathway, the Ovarian Function, and Regulation of Fertility. *Endocrinology.* 2016;157(10):3719-3730.
15. Malki S, Tharp ME, Bortvin A. A Whole-Mount Approach for Accurate Quantitative and Spatial Assessment of Fetal Oocyte Dynamics in Mice. *Biol Reprod.* 2015;93(5):113.
